# Supplementary material for: Pyruvate Dehydrogenase Kinase 1 inhibition mediated oxidative phosphorylation enhancement in cartilage promotes osteoarthritis progression
Source: BMC Musculoskelet Disord. 2023 Jul 20;24:597. doi: 10.1186/s12891-023-06585-6 (PMC10357736; doi:10.1186/s12891-023-06585-6)
Supplement: Supplementary file 1 — Additional file 1. [file 12891_2023_6585_MOESM1_ESM.zip › KEGG.Gsea.1653622432464/KEGG_PENTOSE_AND_GLUCURONATE_INTERCONVERSIONS.html]

Details for gene set KEGG\_PENTOSE\_AND\_GLUCURONATE\_INTERCONVERSIONS[GSEA]

|  || Dataset | 55457\_collapsed\_to\_symbols.cls.cls #OA\_versus\_normal.cls.cls #OA\_versus\_normal\_repos |
| Phenotype | cls.cls#OA\_versus\_normal\_repos |
| Upregulated in class | OA |
| GeneSet | KEGG\_PENTOSE\_AND\_GLUCURONATE\_INTERCONVERSIONS |
| Enrichment Score (ES) | 0.33573842 |
| Normalized Enrichment Score (NES) | 1.0222124 |
| Nominal p-value | 0.40130153 |
| FDR q-value | 0.5721739 |
| FWER p-Value | 1.0 |
Table: GSEA Results Summary

  

Fig 1: Enrichment plot: KEGG\_PENTOSE\_AND\_GLUCURONATE\_INTERCONVERSIONS      
 Profile of the Running ES Score & Positions of GeneSet Members on the Rank Ordered List

  

| SYMBOL | TITLE | RANK IN GENE LIST | RANK METRIC SCORE | RUNNING ES | CORE ENRICHMENT || 1 | UGT2B4 | UDP glucuronosyltransferase family 2 member B4 [Source:HGNC Symbol;Acc:HGNC:12553] | 566 | 0.634 | 0.0945 | Yes |
| 2 | GUSB | glucuronidase beta [Source:HGNC Symbol;Acc:HGNC:4696] | 904 | 0.541 | 0.1864 | Yes |
| 3 | UGT2B17 | UDP glucuronosyltransferase family 2 member B17 [Source:HGNC Symbol;Acc:HGNC:12547] | 2050 | 0.356 | 0.1753 | Yes |
| 4 | UGT2A3 | UDP glucuronosyltransferase family 2 member A3 [Source:HGNC Symbol;Acc:HGNC:28528] | 2854 | 0.265 | 0.1708 | Yes |
| 5 | AKR1B1 | aldo-keto reductase family 1 member B [Source:HGNC Symbol;Acc:HGNC:381] | 3138 | 0.236 | 0.2004 | Yes |
| 6 | RPE | ribulose-5-phosphate-3-epimerase [Source:HGNC Symbol;Acc:HGNC:10293] | 3153 | 0.235 | 0.2506 | Yes |
| 7 | CRYL1 | crystallin lambda 1 [Source:HGNC Symbol;Acc:HGNC:18246] | 3292 | 0.221 | 0.2882 | Yes |
| 8 | UGT1A8 | UDP glucuronosyltransferase family 1 member A8 [Source:HGNC Symbol;Acc:HGNC:12540] | 3301 | 0.221 | 0.3357 | Yes |
| 9 | UGT1A10 | UDP glucuronosyltransferase family 1 member A10 [Source:HGNC Symbol;Acc:HGNC:12531] | 4850 | 0.083 | 0.2338 | No |
| 10 | XYLB | xylulokinase [Source:HGNC Symbol;Acc:HGNC:12839] | 7054 | -0.084 | 0.0811 | No |
| 11 | UGT2B11 | UDP glucuronosyltransferase family 2 member B11 [Source:HGNC Symbol;Acc:HGNC:12545] | 7270 | -0.100 | 0.0864 | No |
| 12 | DCXR | dicarbonyl and L-xylulose reductase [Source:HGNC Symbol;Acc:HGNC:18985] | 7549 | -0.123 | 0.0916 | No |
| 13 | UGP2 | UDP-glucose pyrophosphorylase 2 [Source:HGNC Symbol;Acc:HGNC:12527] | 8735 | -0.211 | 0.0458 | No |
| 14 | UGT2A1 | UDP glucuronosyltransferase family 2 member A1 complex locus [Source:HGNC Symbol;Acc:HGNC:12542] | 9451 | -0.269 | 0.0490 | No |
| 15 | UGDH | UDP-glucose 6-dehydrogenase [Source:HGNC Symbol;Acc:HGNC:12525] | 10279 | -0.349 | 0.0610 | No |
| 16 | UGT2B15 | UDP glucuronosyltransferase family 2 member B15 [Source:HGNC Symbol;Acc:HGNC:12546] | 12225 | -0.652 | 0.0524 | No |
Table: GSEA details [plain text format]

  

Fig 2: KEGG\_PENTOSE\_AND\_GLUCURONATE\_INTERCONVERSIONS      
 Blue-Pink O' Gram in the Space of the Analyzed GeneSet

  

Fig 3: KEGG\_PENTOSE\_AND\_GLUCURONATE\_INTERCONVERSIONS: Random ES distribution      
 Gene set null distribution of ES for **KEGG\_PENTOSE\_AND\_GLUCURONATE\_INTERCONVERSIONS**
